# Long-term Outcomes of Childhood Family Income Supplements on Adult Functioning

**DOI:** 10.1001/jamapediatrics.2022.2946

**Published:** 2022-08-22

**Authors:** William E. Copeland, Guangyu Tong, Lauren Gaydosh, Sherika N. Hill, Jennifer Godwin, Lilly Shanahan, E. Jane Costello

**Affiliations:** 1 Department of Psychiatry, University of Vermont, Burlington; 2 Department of Biostatistics, Yale University, New Haven, Connecticut; 3 Department of Sociology, University of Texas at Austin; 4 Sanford School of Public Policy, Duke University, Durham, North Carolina; 5 Center for Child and Family Policy, Duke University, Durham, North Carolina; 6 Emeritus Faculty, Duke University Medical Center, Durham, North Carolina; 7 Jacobs Center for Productive Youth Development, Department of Psychology, University of Zurich, Zurich, Switzerland

## Abstract

**Importance:**

During an ongoing longitudinal cohort study, a casino opening created a natural cash transfer experiment. Some participating families received income supplements, and others did not. The children in this study are now adults.

**Objective:**

To assess the long-term outcomes of family income supplements received in childhood.

**Design, Setting, and Participants:**

This community-representative longitudinal cohort study set in western North Carolina assessed 1266 participants aged 9, 11, and 13 years at intake up to 11 times up to age 30 years from January 1993 to December 2015. Data were analyzed from January to December 2021.

**Exposures:**

In 1996, a southeastern American Indian tribe implemented a cash transfer program of approximately $5000 annually per person for tribal members. Participants were compared on whether their family ever received the cash transfers (American Indian vs non–American Indian), the duration of the transfers, and annual amount based on the number of parents.

**Main Outcomes and Measures:**

Participants were followed up at ages 25 and 30 years to assess mental health symptoms, substance use symptoms, and functional outcomes (physical health, risky or illegal behaviors, and financial and social functioning).

**Results:**

Of 1266 included participants, 320 (25.3%) were American Indian and 581 (49.7%) were female. Participants whose families received cash transfers during childhood reported fewer anxiety symptoms (relative risk [RR], 0.33; 95% CI, 0.25-0.44), depressive symptoms (RR, 0.51; 95% CI, 0.42-0.62), and cannabis symptoms (RR, 0.47; 95% CI, 0.27-0.82). They also reported improved physical health (RR, 0.66; 95% CI, 0.55-0.80) and financial functioning (RR, 0.78; 95% CI, 0.67-0.89) and fewer risky or illegal behaviors (RR, 0.57; 95% CI, 0.46-0.72) compared with those who did not receive the cash transfer. This pattern was supported by a series of heterogeneity analyses in which children whose families received the transfers for the longest duration and whose families received the largest transfer (due to having multiple American Indian parents) had the lowest levels of symptoms and the highest levels of functioning.

**Conclusions and Relevance:**

In this natural experiment, a family cash transfer in childhood was associated with positive adult functioning 20 years later. The findings support programs like the child tax credit or universal basic income that provide cash directly to families with children.

## Key Points

**Question:**

Is a family cash transfer associated with improved functioning after children have reached adulthood?

**Findings:**

In this cohort study including 1266 participants, participants whose families received cash transfers during their childhood reported fewer anxiety, depressive, and cannabis-related symptoms and improved functioning (physical health and financial well-being) in adulthood compared with participants whose families did not receive a transfer. Benefits were proportional to the cumulative amount of cash the participants’ families received.

**Meaning:**

In this natural experiment, a family cash transfer in childhood was associated with positive adult functioning 20 years later.

## Teaser

This cohort study assesses the long-term outcomes of family income supplements received in childhood.

## Introduction

Human capital investments made in the early life course have the opportunity to accumulate positive benefits across the life span.^1,2^ This logic has spurred interest, both in the research and public policy spheres, in programs like the child tax credit and universal basic income, which boost the financial resources of families with children through direct cash transfers.^3^ The expectations for such programs have been buttressed by results from the Great Smoky Mountains Study (GSMS), a natural experiment in the southeastern US that studied the outcomes of an exogenous income supplement to families of school-aged children on child educational and mental health outcomes.^4^ Because the study began 4 years prior to the implementation of income supplements (ie, a casino opening), the study was able to adjust for preexisting differences between those who received vs did not receive the cash transfer. Positive associations were sustained to age 21 years.^5,6,7,8^ The GSMS now has data available on mental health, substance use, and real-world functioning to age 30 years, which are the focus of this study.

The natural experiment studied here was not designed as a cash transfer experiment. Rather, in 1996 a southeastern American Indian tribe opened a casino on their land grant territory in western North Carolina. Every man, woman, and child who is legally a tribal member, whatever their age or work status, received a percentage of the casino’s profits, paid twice annually. Children’s funds were paid into a trust fund until they graduated high school. The payment varied with the profitability of the casino; on average, it was at least $5000 per person each year. This increase in income was not causally related to characteristics of the participants or their families (apart from the entire intervention group being American Indian). This is a feature shared by universal basic income programs. Non–American Indian families living in the counties surrounding the reservation did not receive income supplements and served as a comparison group here.

The study is also able to test heterogeneity in the cash transfers within the American Indian group. First, this study has a cohort-sequential design with participants aged 9, 11, and 13 years at baseline. This provides an opportunity to compare participants based on the duration of time that their families received the transfers while participants lived at home. Second, the amount of cash that the families received was dependent on the number of American Indian parents living in the home. The present study tests whether the cash transfer continued to affect outcomes in adulthood and whether the greatest benefits were observed among those participants whose families received the supplement the longest and who received the largest transfer due to having multiple American Indian parents.

## Methods

### Participants

The GSMS is a longitudinal, community-representative study of 1420 children aged 9, 11, and 13 years in 1993. They were recruited from 11 contiguous counties in the Appalachian mountains of western North Carolina. A tribe located in the study area agreed to permit the study to recruit all tribal members aged 9, 11, and 13 years attending schools on the reservation and public schools in the 11-county area. A total of 450 American Indian children were identified; 349 (81%) agreed to participate in the study. This study followed the Strengthening the Reporting of Observational Studies in Epidemiology (STROBE) reporting guidelines.^9^ This study was approved by the Duke University Medical Center ethical review board, and all participants provided written informed consent.

Non–American Indian children were randomly selected from school files from a population of approximately 20 000 school-aged children, based on records from all public schools in the area, using a household equal-probability design. Next, participants were screened for risk of psychopathology; participants screening at high risk were oversampled to allow for study of less common mental health conditions in addition to a random sample of the rest for a total of 1070 non–American Indian children. See the eFigure in the Supplement and studies by Costello et al^6,10^ and Copeland et al^11^ for additional detail. Sampling weights were applied to adjust for differential probability of selection and to allow results to generalize to the broader population.

Annual assessments were completed with the participants and their primary caregivers until participants were aged 16 years; thereafter, participants were interviewed alone at ages 19, 21, 25, and 30 years (data from ages 25 and 30 years are used for this analysis). A total of 11 233 of 13 434 possible interviews (83%) have been completed; by age 30 years, 39 participants had died. Before interviews, all participants and their parents (up to age 18 years) signed informed consent forms. Participants received compensation for their time ($100 for most recent waves). The study protocol was the same for American Indian and non–American Indian participants.

### Preexposure Variables

The study collected data during the 4 years prior to the casino opening. Thus, participant and family characteristics were assessed thoroughly prior to the onset of the cash transfers (or knowledge of the upcoming cash transfer). All variables were assessed through parent and child interviews using the Child and Adolescent Psychiatric Assessment (CAPA).^12^ Race and ethnicity were assessed via parent report and included African American, American Indian, Asian, Hispanic, White, and other race. Measured family characteristics included family structure, family income, and low socioeconomic status. The latter was determined from whether the family met 2 of 3 indicators: low parental educational attainment (parents did not graduate from high school), household poverty status according to federal guidelines, and parental occupational status in bottom 25% on NORC General Social Survey occupational prestige scale (eg, clerk, grocery bagger).^13^ Measured child variables included sex, low birth weight, body mass index, and symptoms of common mental health problems, including anxiety symptoms, depressive symptoms, and behavioral symptoms. Symptoms were coded if reported by either the parent or the child. For mental health symptoms, interviews focused on the 3-month period immediately preceding the interview to maximize accuracy and minimize forgetting and recall bias.^14,15,16^ The reliability and validity of the CAPA for assessing psychiatric symptoms is good to excellent and similar to other diagnostic interviews.^17,18,19^ Adjustment for the preexposure variables allows for a test of the cash transfer independent of baseline characteristics.

### Adult Outcomes

Adult functioning was assessed with psychiatric symptoms, substance use–related symptoms, and adult functioning in important life domains at ages 25 and 30 years. All adult outcomes, except where noted (eg, official criminal records), were assessed using the Young Adult Psychiatric Assessment,^20^ an upward extension of the CAPA interview administered. Outcomes were aggregated across the 2 observations using an either/or rule such that each participant only provided 1 record for the analyses (eg, if a participant met criteria for an anxiety disorder at either age 25 or 30 years, adult anxiety disorder was coded positive). The assessment of adult psychiatric status resembled that of childhood disorders but relied on self-report only. Psychiatric and substance functioning included count variables of symptoms of any *DSM*-*5* anxiety, depressive, alcohol, and cannabis use disorder. Everyday functioning was assessed with 4 scales characterizing physical health, risky or illegal behavior, financial functioning, and social functioning. These scales were summed from relatively common dichotomous indicators in each domain (eg, college completion for financial functioning, smoking status for physical health). In some cases, indicators were coded positive if reported at either adult observation; in other cases (eg, educational attainment), the last observation was used to determine status. All scales are scored such that higher scores indicate worse functioning. Standardized scale scores were obtained by subtracting the individual score from the group mean and dividing the resultant score by the standard deviation. A full description of all indicators used to construct these scales is available in the eMethods in the Supplement.

### Statistical Analysis

Weighted quasi-Poisson regression models (with a log link) tested associations between ever receiving the family cash transfer, which is equivalent with American Indian status in this natural experiment, on each adult outcome. All models adjust for family and individual characteristics prior to the opening of the casino, including baseline status of the outcome variables (eg, anxiety symptoms).

Three sets of additional models examined this heterogeneity in relation to the adult outcomes. The first tested whether children whose families received the benefit the longest displayed more positive adult outcomes. This was done by including a variable that counted the number of years during which the family received the cash transfer before the child reached age 18 years: 6 years for youngest American Indian cohort, 4 years for the middle cohort, 2 years for the oldest cohort, and 0 years for the non–American Indian participants. The next model tested whether any associations differed by its dosage, which was indexed by the number of American Indian parents in the home. If the cash transfer was $5000 in a given year, then households with 2 American Indian parents received approximately $10 000 that year, those with 1 American Indian parent received $5000, and those with no American Indian parents received $0. These follow-up analyses help clarify whether any beneficial associations of the cash transfer were attributable in part to (1) the duration of the supplement exposure in childhood and (2) the amount of the supplement based on the number of American Indian parents. A third analysis tested a variable that combined information about the duration (0, 2, 4, or 6 years for non–American Indian groups and the oldest, the middle, and the youngest American Indian cohort, respectively) and dose of the supplement due to the number of American Indian parents (0, 1, or 2) into a single variable, with a range from 0 to 12. With an average transfer of $5000, this equates to a range of $60 000 in how much cash families received.

A total of 1266 of the living 1381 participants (91.7%) were followed-up with at ages 25 or 30 years. American Indian status was not associated with lower levels of participation in adulthood, suggesting no differential dropout. Ten complete data sets were imputed to address potential missingness in both outcomes and covariates.^21^

All percentages provided are weighted and sample sizes are unweighted. Results from regression models are exponentiated, and relative risks (RRs) and 95% CIs are presented. Findings are considered statistically significant at *P* < .05, and all *P* values were 2-tailed. Analyses were conducted using R version 4.0.1 (The R Foundation). The mice package (multivariate imputation by chained equations) in R was used for both the imputation and synthesis of results in regression analysis.

## Results

Of 1266 included participants, 320 (25.3%) were American Indian and 581 (49.7%) were female. Table 1 provides descriptive information for the American Indian and non–American Indian subsamples, including childhood and family functioning prior to the opening of the casino. Compared with non–American Indian youth, American Indian youth had more family-level and child-level risk factors in several areas, including low socioeconomic status, more family instability, and higher body mass index. American Indian youth had fewer depressive symptoms and more conduct symptoms than non–American Indian youth. There were no differences between the groups on anxiety or oppositional defiant symptoms. There was minimal evidence of differences between the American Indian cohorts prior to the onset of the cash transfer (eTable 1 in the Supplement).

**Table 1.** Descriptive Statistics Prior to Opening of Casino

Characteristic	No. (%)^a^		*P* value
	Non–American Indian^b^	American Indian^b^	
Age cohort group			
Youngest (age 9 y)	378 (36.4)	110 (34.2)	.95
Middle (age 11 y)	356 (34.9)	117 (36.7)	
Oldest (age 13 y)	296 (28.7)	92 (29.1)	
Number of American Indian parents			
2	0	185 (58.8)	<.001
1	6 (0.4)	119 (36.7)	
0	1024 (99.6)	15 (4.5)	
Sex			
Female	424 (49.7)	157 (49.1)	.96
Male	522 (50.2)	163 (50.9)	
Childhood risk factors			
Low socioeconomic status	344 (25.9)	171 (54.3)	<.001
Family instability	227 (17.4)	98 (30.7)	.02
Low birth weight (<2500 g)	63 (7.0)	13 (4.5)	.53
Income level, mean (SD)^c^	6.24 (3.64)	3.70 (2.82)	<.001
Body mass index, mean (SD)^d^	20.73 (4.47)	24.83 (7.63)	<.001
Childhood symptoms, mean (SD)^e^			
Depression	0.78 (0.68)	0.65 (0.62)	.002
Anxiety	0.98 (1.27)	0.89 (1.17)	.29
Oppositional disorder	0.67 (0.86)	0.66 (0.98)	.55
Conduct disorder	0.35 (0.62)	0.46 (0.71)	.02

^a^ Numbers are unweighted, and percentages and means are weighted.

^b^ Race and ethnicity were assessed via parent report and included African American, American Indian, Asian, Hispanic, White, and other race.

^c^ Income levels were coded as follows: 0 indicates no income; 1, $1 to $5000; 2, $5001 to $10 000; 3, $10 001 to $15 000; 4, $15 001 to $20 000; 5, $20 001 to $25 000; 6, $25 001 to $30 000; 7, $30 001 to $35 000; 8, $35 001 to $40 000; 9, $40 001 to $45 000; 10, $45 001 to $50 000; 11, $50 001 to $55 000; 12, $55 001 to $60 000; and 13, more than $60 000.

^d^ Calculated as weight in kilograms divided by height in meters squared.

^e^ Childhood symptoms were coded as total symptom counts for each diagnostic category.

The first model tested whether ever having received the cash transfer was associated with adult mental health, substance, and functional outcomes (Table 2). These analyses adjusted for sex, cohort, and covariates prior to the casino opening, including low socioeconomic status, family instability, household income, low birth weight, body mass index, and anxiety, depressive, and behavioral symptoms. Participants whose families received cash transfers during childhood reported fewer anxiety symptoms (RR, 0.33; 95% CI, 0.25-0.44), depressive symptoms (RR, 0.51; 95% CI, 0.42-0.62), and cannabis symptoms (RR, 0.47; 95% CI, 0.27-0.82). Those receiving the transfer also reported improved physical health (RR, 0.66; 95% CI, 0.55-0.80) and financial functioning (RR, 0.78; 95% CI, 0.67-0.89) and fewer risky or illegal behaviors (RR, 0.57; 95% CI, 0.46-0.72) compared with those who did not receive the cash transfer. Overall, the findings are consistent with a broad-based pattern of positive outcomes for cash transfer participants.

**Table 2.** Association of Ever Receiving the Family Cash Transfer With Adult Mental Health, Substance, and Functional Outcomes^a^

Outcome	Mean (SD)^b^		Relative risk (95% CI)
	Received cash transfer	Did not receive cash transfer	
Adult psychiatric			
Anxiety symptoms	0.58 (1.42)	1.94 (2.55)	0.33 (0.25-0.44)^c^
Depression symptoms	0.76 (1.11)	1.57 (1.11)	0.51 (0.42-0.62)^c^
Adult substance			
Alcohol symptoms	0.40 (1.14)	0.48 (1.20)	0.97 (0.52-1.81)
Cannabis symptoms	0.18 (0.67)	0.34 (1.12)	0.47 (0.27-0.82)^c^
Adult functional			
Physical health	0.23 (0.74)	0 (1.00)	0.66 (0.55-0.80)^c^
Financial/employment	0.14 (0.85)	0.01 (1.00)	0.78 (0.67-0.89)^c^
Risky/illegal behavior	0.34 (0.79)	0 (0.99)	0.57 (0.46-0.72)^c^
Social relationships	0.06 (0.98)	0 (1.00)	1.04 (0.88-1.21)

^a^ All results are combined across 10 imputed data sets using the Rubin formula. All models adjusted for the following childhood preexposure measures: depression symptoms, anxiety symptoms, sum of oppositional disorder and conduct disorder symptoms, cohort, sex, low socioeconomic status, family instability, low birth weight, household income, body mass index, and body mass index squared.

^b^ Symptoms were coded as total symptom counts for each diagnostic category.

^c^ *P* < .05.

The next set of models tested associations based on the duration of the cash transfer when the child lived with their family (0, 2, 4, or 6 years) and annual amount of cash received by the family indexed by the number of American Indian parents in the home (0, 1, or 2 parents). The first set of columns in Table 3 tested the duration of cash transfers for all of the adult outcomes. Longer duration of exposure to the cash transfer in childhood was associated with reductions in anxiety, depressive, and cannabis symptoms. For the functional outcomes, the duration of exposure was associated with improved physical health and financial functioning as well as decreased risky or illegal behaviors. An additional set of analyses compared each of the American Indian cohorts individually with the non–American Indian cohorts and with one another (eTables 2 and 3 in the Supplement). The next set of columns in Table 3 tested the annual amount of cash received by the family. Having more American Indian parents in the home was associated with reduced anxiety, depressive, and cannabis symptoms; improved physical health and financial functioning; and lower levels of risky or illegal behaviors. An additional set of analyses compared those with 2 American Indian parents or 1 American Indian parent with those with no American Indian parents and with one another (eTables 4 and 5 in the Supplement). Finally, the last set of columns in Table 3 look at the product of duration and annual amount due to the number of American Indian parents or the cumulative exposure to the cash transfer within childhood (range from 0 to 12). The results were largely consistent with previous analyses, suggesting that increased exposure to the cash transfer were associated with lower levels of psychiatric symptoms, improved physical health and financial functioning, and lower levels of risky or illegal behaviors.

**Table 3.** Associations of Duration of Exposure to Transfer, Annual Amount, and Cumulative Exposure With Adult Outcomes^a^

Outcomes	Relative risk (95% CI)		
	Cash transfer duration (0, 2, 4, or 6 y)	American Indian parents (0, 1, or 2)	Cumulative exposure^b^
Adult psychiatric			
Anxiety symptoms	0.78 (0.72-0.84)^c^	0.51 (0.42-0.63)^c^	0.85 (0.81-0.89)^c^
Depression symptoms	0.87 (0.83-0.92)^c^	0.65 (0.58-0.74)^c^	0.91 (0.89-0.94)^c^
Adult substance			
Alcohol symptoms	1.01 (0.88-1.16)	0.97 (0.66-1.42)	1.01 (0.93-1.10)
Cannabis symptoms	0.84 (0.73-0.97)^c^	0.57 (0.40-0.81)^c^	0.89 (0.81-0.97)^c^
Adult functional			
Physical health	0.93 (0.88-0.97)^c^	0.75 (0.66-0.84)^c^	0.95 (0.93-0.98)^c^
Financial/employment	0.96 (0.92-0.99)^c^	0.86 (0.79-0.94)^c^	0.98 (0.96-1.00)^c^
Risky or illegal behavior	0.90 (0.85-0.95)^c^	0.70 (0.61-0.81)^c^	0.94 (0.90-0.97)^c^
Social relationships	1.03 (0.99-1.07)	1.03 (0.94-1.13)	1.02 (1.00-1.04)

^a^ All results are combined across 10 imputed data sets using the Rubin formula. All results based on linear regression models adjusting for sex, cohort, and the following covariates prior to the casino opening: family low socioeconomic status, family instability, household income, low birth weight, body mass index, anxiety symptoms, depressive symptoms, and behavioral symptoms.

^b^ Combination of information about the duration (0, 2, 4, or 6 years for non–American Indian groups and the oldest, the middle, and the youngest American Indian cohort, respectively) and dose of the cash supplement due to the number of American Indian parents (0, 1, or 2), with a range from 0 to 12.

^c^ *P* < .05.

Finally, models tested in Table 2 and Table 3 were reanalyzed using only information from the age 30 years assessment (eTables 6 and 7 in the Supplement) to test for attenuation. Results were largely the same as has been reported above.

## Discussion

In this cohort study, close to 20 years after a cash transfer to families began, the children (now adults) had fewer mental health problems and improved functional outcomes than their non–American Indian peers despite having started out with fewer financial resources during their first decade. These improvements were proportional to the cumulative amount of cash the participants’ families received while the child lived at home. Our cautious conclusion is that the relief from financial stress and additional resources brought by the extra income were beneficial to families and their children. Even at age 30 years, long after leaving home and accomplishing select milestones of adult life (eg, marriage, children), the benefits of the childhood cash transfer for participants’ well-being persisted.

There are alternative explanations for the positive outcomes in the American Indian participants that merit consideration. First, the positive outcomes may be explained by other investments made by the tribe in the community to improve local resources, like health care, education, and housing. Indeed, although half of the proceeds from the casino were set aside to support direct cash payments to tribal members, the other half was used for community investments.^22^ Second, the observed findings could reflect cash transfers the participants received as adults rather than the cash transfers their parents received when they were children. At age 18 years, the American Indian study participants received escrow payouts from the funds they accumulated as children and then received the biannual payments themselves going forward. Third, the associated economic benefits associated with the casino may have protected American Indian participants from an increase in despair, which was observed in adjacent Appalachian economically distressed communities.^22,23^ Finally, a number of these factors together may have contributed to an increase in the social cohesion of the community itself.^24^

Each of these explanations is plausible and consistent with the main effects analysis, yet none fit with the pattern of findings in the follow-up analyses. Here, the beneficial associations of the cash transfer were dependent on the different exposures to the family income supplement during childhood, in terms of duration, number of American Indian parents, and their cumulative effect. This means, for example, that beneficial associations of the cash transfers are not because of receiving the cash transfers as adults, as all American Indian participants have had the same exposure within adulthood. Overall, the pattern of results suggests that a significant portion of the beneficial association is related to the cumulative income supplement received by participants’ families during childhood.

This study is only possible because of unlikely, fortuitous circumstances: a natural experiment occurring 4 years into an ongoing study that has continued for 2 decades hence. While the positive associations were consistently observed over time, the specific benefit was different in each developmental period to date. In the first 4 years of the experiment, the observed benefit during childhood was on behavioral symptoms and delinquency.^4^ When participants were young adults, the beneficial associations were specific to substance disorders involving alcohol and cannabis.^5^ Now, in adulthood, the participants display improved emotional health and benefits in important life areas, like physical health and criminal behavior. These apparent shifts closely track the key issues that young people have to master in each of these developmental phases.^25,26^ What is surprising from a developmental perspective is not the particular pattern of the benefits but its persistence for 2 decades and, in particular, the differences related to cumulative family supplement exposure.

### Limitations

The study has a number of limitations to consider. First, the participants were not randomly assigned to the cash transfer condition; thus, unmeasured confounding that could affect the results, such as variations in neurodevelopmental stage. Second, race and ethnicity were confounded with the intervention, although it was possible to test heterogeneity related to duration and total annual amount of cash transfer exposure. Similarly, the improved outcomes related to total annual amount of cash transfer were confounded with the number of parents in the home. Additionally, the study took place in a primarily rural area of the southeast US, and results may not generalize to other communities.

## Conclusion

There is currently great interest in efforts to reduce childhood poverty via expansion of the child tax credit as well as proposals for a universal basic income. Such proposals based on unconditional transfers have often been critiqued as inefficient compared with targeted assistance. While this intervention did not specifically target families based on economic need, the census tracts that make up the GSMS are generally rated as low or moderate in terms of childhood opportunity levels (including those areas where most tribal members live).^27^ Overall, these findings demonstrate the persisting merits of a simple system that provides parents with the resources to foster the health and functioning of their children as best they see fit.

## Notes

Supplement.
eMethods.
**eTable 1.** Comparison of American Indian Cohorts Prior to Casino Opening **eTable 2.** Comparison of Individual American Indian Cohorts With Non–American Indian Cohorts **eTable 3.** Comparison Between Individual American Indian Cohorts **eTable 4.** Comparison of 2 American Indian Parents Group and 1 American Indian Parent Group With the No American Indian Parents Group **eTable 5.** Comparison Between 2 American Indian Parents Group and 1 American Indian Parent Group **eTable 6.** Association of Ever Receiving the Family Cash Transfer With Adult Mental Health, Substance, and Functional Outcomes (Age 30 Years Outcomes Only) **eTable 7.** Associations of Duration of Exposure to Transfer, Annual Amount, and Cumulative Exposure With Adult Outcomes (Age 30 Years Outcomes Only) **eFigure.** Ascertainment of the Original Great Smoky Mountains Study Sample
