# Supplementary material for: Long-term Outcomes of Childhood Family Income Supplements on Adult Functioning
Source: JAMA Pediatr. 2022 Aug 22;176(10):1020–6. doi: 10.1001/jamapediatrics.2022.2946 (PMC9396462; doi:10.1001/jamapediatrics.2022.2946)
Supplement: Supplement. — eMethods. eTable 1. Comparison of American Indian Cohorts Prior to Casino Opening eTable 2. Comparison of Individual American Indian Cohorts With Non–American Indian Cohorts eTable 3. Comparison Between Individual American Indian Cohorts eTable 4. Comparison of 2 American Indian Parents Group and 1 American Indian Parent Group With the No American Indian Parents Group eTable 5. Comparison Between 2 American Indian Parents Group and 1 American Indian Parent Group eTable 6. Association of Ever Receiving the Family Cash Transfer With Adult Mental Health, Substance, and Functional Outcomes (Age 30 Years Outcomes Only) eTable 7. Associations of Duration of Exposure to Transfer, Annual Amount, and Cumulative Exposure With Adult Outcomes (Age 30 Years Outcomes Only) eFigure. Ascertainment of the Original Great Smoky Mountains Study Sample [file jamapediatr-e222946-s001.pdf]

Supplemental Online Content

Copeland WE, Tong G, Gaydosh L, et al. Long-term outcomes of childhood family income supplements on adult functioning. *JAMA Pediatr*. Published online August 22, 2022. doi:10.1001/jamapediatrics.2022.2946

eMethods.
eTable 1. Comparison of American Indian Cohorts Prior to Casino Opening
eTable 2. Comparison of Individual American Indian Cohorts With Non–American Indian Cohorts
eTable 3. Comparison Between Individual American Indian Cohorts
eTable 4. Comparison of 2 American Indian Parents Group and 1 American Indian Parent Group With the No American Indian Parents Group
eTable 5. Comparison Between 2 American Indian Parents Group and 1 American Indian Parent Group
eTable 6. Association of Ever Receiving the Family Cash Transfer With Adult Mental Health, Substance, and Functional Outcomes (Age 30 Years Outcomes Only)
eTable 7. Associations of Duration of Exposure to Transfer, Annual Amount, and Cumulative Exposure With Adult Outcomes (Age 30 Years Outcomes Only)
eFigure. Ascertainment of the Original Great Smoky Mountains Study Sample

This supplementary material has been provided by the authors to give readers additional information about their work.

© 2022 Copeland WE et al. *JAMA Pediatrics*.

eMethods.

Adult functional Outcomes:

Health. Participants reported being *diagnosed with a serious illness* or being in a *serious accident* at any point during young adulthood. Having a *sexually transmitted disease* was coded if the participant reported testing positive for herpes, genital warts, chlamydia, or HIV. At each assessment, weight and height measurements were used to derive body mass index. *Obesity* was defined by CDC definitions up to age 20 and greater than or equal to 30 kg/m^2 thereafter. Participants were assessed for a *DSM-IV psychiatric diagnosis* including the following: any DSM-IV anxiety disorder, any depressive disorder, and antisocial personality disorder. *Regular smoking* was defined as smoking > 1 cigarette per day for 3 months. A physical health problems survey adapted from Form HIS-1A (1998), US Department of Commerce for the U.S. Public Health Service was administered at all interviews. This assessed self-reported *perceived poor health*, *high illness contagion risk*, and *slow illness recovery*. Finally, a dichotomous variable was included to indicate if participant had a C-Reactive protein level of greater than 3.0, which is considered a risk factor for cardiovascular disease.

Risky/illegal behaviors. Official felony charges were harvested from NC administrative Offices of the Courts records. Self-report was used to assess recent *police contact*, often *lying* to others, *frequent physical fighting*, and *breaking into another home/business/property*. Frequent *drunkenness* was coded if subject reported drinking to excess at least once weekly for 3 months. Subjects reported recent use of *marijuana* or other *illegal substances*. Finally, subjects reported having one-time sexual encounters with strangers (*hooking up with strangers*).

Wealth: Financial/educational accomplishments. *Being impoverished* was coded based on thresholds issued by the Census Bureau based on income and family size [36]. *High school*

dropout and *completion of any college education* were coded based upon the subject's educational status at the last adult assessment. The following job problems were assessed at each assessment: *being dismissed or fired* from a job and *quitting a job* without financial preparations. Finally, other financial problems assessed included: *failing to honor debts or financial obligations* and being *a poor manager of one's finances*.

Social relationships. *Marital, parenthood,* and *divorce* status were determined through self-report at the last adult assessment. The quality of the participant's relationship with their parents, spouse/significant other, and friends was assessed at each assessment including arguments and violence. Variables were included to indicate any *violence in a primary relationship*, *poor relationship with one's parents*, *no best friend or confidante*, and *problems making or keeping friends*.

© 2022 Copeland WE et al. *JAMA Pediatrics*.

eTable 1. Comparison of American Indian Cohorts Prior to Casino Opening

	**Youngest Cohort**	**Middle Cohort**	**Oldest Cohort**	**P value**
	**M (SD) of n (%)**	**M (SD) of n (%)**	**M (SD) of n (%)**	
**Demographics**				
#AI Parents				
Two	62(57.9%)	68(58.3%)	55(60.4%)	0.5740
One	44(39.3%)	41(34.8%)	34(36.3%)	
Zero	4(2.8%)	8(7.0%)	3(3.3%)	
Male	57(51.4%)	59(49.6%)	47(50.5%)	0.9632
**Childhood risk factors**				
Low SES	62(57.9%)	66(56.5%)	43(47.3%)	0.2695
Family Instability	33(30.0%)	41(35.7%)	24(26.4%)	0.3202
Low Birthweight	6(5.8%)	6(5.6%)	1(1.3%)	0.0193
Income Level	3.43(2.46)	3.45(2.75)	4.48(3.09)	0.0119
Body Mass Index	23.43(8.93)	25.00(6.60)	26.08(6.69)	0.0436
**Childhood health**				
Depression	0.61(0.56)	0.73(0.59)	0.59(0.71)	0.1993
Anxiety	0.90(1.09)	0.98(1.28)	0.72(1.06)	0.2527
Oppositional Disorder	0.59(0.96)	0.81(1.11)	0.52(0.75)	0.0777
Conduct Disorder	0.30(0.45)	0.57(0.81)	0.48(0.76)	0.0114

Note: p values obtained from Chi-square tests for categorical variables and from ANOVA tests for continuous variables.

© 2022 Copeland WE et al. *JAMA Pediatrics*.

eTable 2. Comparison of Individual American Indian Cohorts With Non–American Indian Cohorts

Outcomes	Youngest cohort				Middle cohort				Oldest cohort				Wald equality test
	RR	CI		p	RR	CI		p	RR	CI		p	
**Psychiatric**													
Anxiety sx.	0.27	0.16	0.44	0.0000	0.37	0.23	0.59	0.0000	0.37	0.23	0.60	0.0001	0.0000
Depression sx.	0.40	0.30	0.55	0.0000	0.54	0.40	0.73	0.0001	0.65	0.45	0.92	0.0156	0.0000
**Substance**													
Alcohol sx.	0.72	0.29	1.77	0.4743	1.07	0.51	2.26	0.8503	1.20	0.54	2.65	0.6596	0.7613
Cannabis sx.	0.34	0.13	0.88	0.0267	0.70	0.33	1.49	0.3574	0.33	0.12	0.95	0.0420	0.0364
**Functional**													
Physical health	0.50	0.37	0.66	0.0000	0.75	0.57	0.99	0.0446	0.80	0.59	1.07	0.1342	0.0000
Financial/ employment	0.63	0.49	0.81	0.0006	0.81	0.66	1.00	0.0483	0.92	0.71	1.19	0.5184	0.0029
Risky/illegal	0.43	0.30	0.60	0.0000	0.57	0.41	0.79	0.0007	0.76	0.52	1.12	0.1670	0.0000
Social	0.79	0.62	1.00	0.0542	1.09	0.85	1.41	0.5008	1.34	1.06	1.69	0.0153	0.0065

Note: All results based on linear regression models adjusting for sex, cohort, and the following covariates prior to the casino opening: family low SES status, family instability, household income, low birthweight, body mass index, anxiety symptoms, depressive symptoms, and behavioral symptoms. All results are combined across 10 imputed datasets by Rubin's formula.

© 2022 Copeland WE et al. *JAMA Pediatrics*.

eTable 3. Comparison Between Individual American Indian Cohorts

Outcomes	Youngest vs. Oldest				Middle vs. Oldest Cohort			
	RR	CI		p	RR	CI		p
**Psychiatric**								
Anxiety sx.	0.72	0.36	1.41	0.3314	0.98	0.50	1.92	0.9588
Depression sx.	0.62	0.39	0.99	0.0463	0.83	0.54	1.29	0.4160
**Substance**								
Alcohol sx.	0.60	0.23	1.56	0.2964	0.90	0.36	2.26	0.8201
Cannabis sx.	1.03	0.28	3.74	0.9659	2.11	0.57	7.81	0.2629
**Functional**								
Physical health	0.62	0.43	0.91	0.0140	0.95	0.65	1.37	0.7723
Financial/ employment	0.69	0.47	0.99	0.0469	0.88	0.64	1.21	0.4364
Risky/illegal	0.56	0.34	0.91	0.0203	0.75	0.46	1.22	0.2521
Social	0.59	0.43	0.81	0.0011	0.82	0.58	1.14	0.2390

Note: All results based on linear regression models adjusting for sex, cohort, and the following covariates prior to the casino opening: family low SES status, family instability, household income, low birthweight, body mass index, anxiety symptoms, depressive symptoms, and behavioral symptoms. All results are combined across 10 imputed datasets by Rubin's formula.

© 2022 Copeland WE et al. *JAMA Pediatrics*.

eTable 4. Comparison of 2 American Indian Parents Group and 1 American Indian Parent Group With the No American Indian Parents Group

**Outcomes**	**Two AI parents**				**One AI parent**				**Wald equality test**
	RR	CI		p	RR	CI		p	
**Psychiatric**									
Anxiety sx.	0.24	0.16	0.36	0.0000	0.57	0.35	0.94	0.0263	0.0000
Depression sx.	0.41	0.33	0.52	0.0000	0.70	0.52	0.94	0.0194	0.0000
**Substance**									
Alcohol sx.	1.09	0.52	2.28	0.8155	0.63	0.30	1.34	0.2333	0.4067
Cannabis sx.	0.31	0.15	0.62	0.0012	0.60	0.31	1.17	0.1344	0.0030
**Functional**									
Physical health	0.58	0.46	0.72	0.0000	0.70	0.52	0.95	0.0223	0.0000
Financial/ employment	0.79	0.67	0.92	0.0036	0.74	0.60	0.91	0.0043	0.0014
Risky/illegal	0.54	0.41	0.71	0.0000	0.58	0.39	0.84	0.0009	0.0000
Social	1.04	0.87	1.26	0.6543	1.06	0.89	1.26	0.4853	0.7832

Note: All results based on linear regression models adjusting for sex, cohort, and the following covariates prior to the casino opening: family low SES status, family instability, household income, low birthweight, body mass index, anxiety symptoms, depressive symptoms, and behavioral symptoms. All results are combined across 10 imputed datasets by Rubin's formula.

eTable 5. Comparison Between 2 American Indian Parents Group and 1 American Indian Parent Group

**Outcomes**	Two AI parents vs. One AI parent			
	RR	CI		p
**Psychiatric**				
Anxiety sx.	0.43	0.23	0.78	0.0054
Depression sx.	0.58	0.41	0.83	0.0029
**Substance**				
Alcohol sx.	1.73	0.76	3.95	0.1915
Cannabis sx.	0.52	0.24	1.11	0.0894
**Functional**				
Physical health	0.82	0.59	1.14	0.2486
Financial/ employment	1.06	0.84	1.34	0.6057
Risky/illegal	0.94	0.66	1.36	0.7561
Social	0.98	0.80	1.20	0.8526

Note: All results based on linear regression models adjusting for sex, cohort, and the following covariates prior to the casino opening: family low SES status, family instability, household income, low birthweight, body mass index, anxiety symptoms, depressive symptoms, and behavioral symptoms. All results are combined across 10 imputed datasets by Rubin's formula.

© 2022 Copeland WE et al. *JAMA Pediatrics*.

eTable 6. Association of Ever Receiving the Family Cash Transfer With Adult Mental Health, Substance, and Functional Outcomes (Age 30 Years Outcomes Only)

		RR	**95% CI**	
**Adult psychiatric**				
Anxiety symptoms		0.30	0.21	0.45
Depression symptoms		0.45	0.33	0.60
**Adult substance**				
Alcohol symptoms		1.02	0.53	1.95
Cannabis symptoms		0.36	0.16	0.83
**Adult functional**				
Physical health		0.82	0.66	1.01
Financial/employment		0.84	0.71	0.98
Risky/illegal behavior		0.59	0.44	0.78
Social relationships		0.94	0.78	1.14

Note: All results are combined across 10 imputed datasets by Rubin's formula. All models adjusted for the following childhood pre-exposure measures: depression sx., anxiety sx., sum of oppositional disorder and conduct disorder sx., cohort, sex, low SES status, family instability, low birthweight, household income, BMI, and BMI^2. Models in which the confidence intervals do not include 0 are significant at $p < 0.05$.

© 2022 Copeland WE et al. *JAMA Pediatrics*.

eTable 7. Associations of Duration of Exposure to Transfer, Annual Amount, and Cumulative Exposure With Adult Outcomes (Age 30 Years Outcomes Only)

Outcomes	Number of Childhood Years with Transfer				Number of AI parents				Cumulative Exposure (Number of AI parents * Years)		
	Relative Risk	95% CI			Relative Risk	95% CI			Relative Risk	95% CI	
**Adult psychiatric**											
Anxiety symptoms	0.78	0.69	0.87		0.50	0.37	0.68		0.84	0.78	0.91
Depression symptoms	0.86	0.79	0.93		0.55	0.46	0.66		0.90	0.85	0.94
**Adult substance**											
Alcohol symptoms	1.02	0.87	1.20		1.05	0.69	1.60		1.04	0.94	1.14
Cannabis symptoms	0.78	0.63	0.98		0.50	0.28	0.91		0.86	0.75	1.00
**Adult functional**											
Physical health	0.98	0.93	1.03		0.87	0.76	0.98		0.99	0.96	1.02
Financial/employment	0.97	0.94	1.01		0.90	0.82	0.99		0.99	0.96	1.01
Risky/illegal behavior	0.91	0.85	0.98		0.72	0.60	0.86		0.95	0.90	0.99
Social relationships	1.01	0.97	1.06		0.98	0.88	1.09		1.01	0.98	1.04

Note: All results are combined across 10 imputed datasets by Rubin's formula. All results based on linear regression models adjusting for sex, cohort, and the following covariates prior to the casino opening: family low SES status, family instability, household income, low birthweight, body mass index, anxiety symptoms, depressive symptoms, and behavioral symptoms. Models in which the confidence intervals do not include 0 are significant at p <0.05.

© 2022 Copeland WE et al. *JAMA Pediatrics*.

eFigure. Ascertainment of the Original Great Smoky Mountains Study Sample

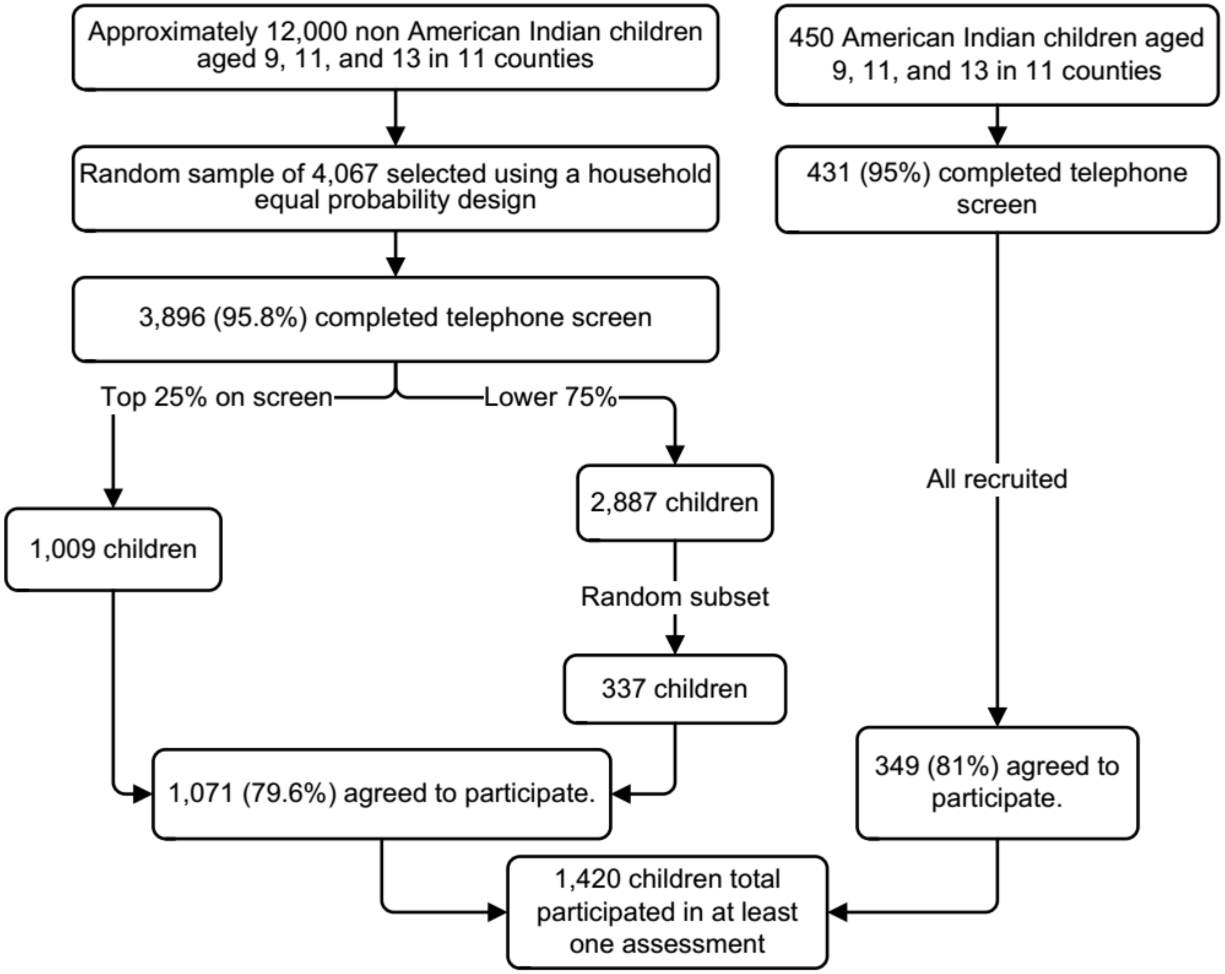

© 2022 Copeland WE et al. *JAMA Pediatrics*.
